# Post-COVID-19 tsunami of periodontal diseases

**DOI:** 10.34172/japid.2021.009

**Published:** 2021-06-09

**Authors:** Adileh Shirmohammadi

**Affiliations:** ^1^Department of Periodontics, Faculty of Dentistry, Tabriz University of Medical Sciences, Tabriz, Iran

Oral hygiene entails cleaning the teeth and the oral cavity to prevent gingival diseases, periodontitis, and halitosis. Oral hygiene measures are necessary and inevitable for everyone throughout life. Halitosis is a medical and social problem in all communities.^1^ Halitosis is defined as a malodor that mainly originates from the oral cavity, which might occur when an individual has poor oral hygiene.^2^ One reason for toothbrushing is to avoid oral malodor during social interactions, indicating the importance of observing oral hygiene measures.^3^

Wearing a mask is mandatory during the COVID-19 pandemic to prevent the spread of the virus through respiratory droplets, negating the commitment to observe oral hygiene measures during social interactions.^4^ Therefore, it results in a decrease in the observation of individual oral hygiene measures. This increases the severity or the odds of developing periodontal disease. In addition, wearing a mask and drinking no liquids at the workplace increase oral mucosal dryness, exacerbating periodontal diseases.^5,6^ Another factor that exacerbates periodontal diseases or results in the recurrence of periodontal diseases is that the treated patients do not attend periodontal maintenance sessions. Periodontal maintenance procedures involve the removal of plaque and calculus and an evaluation process geared toward identifying factors that might interfere with oral health. These visits might be scheduled every few weeks or as far apart as every six months. The dentist determines the frequency of the visits, depending on disease severity, the overall oral health, and the risk factors involved.^7^ Most people with a history of periodontal disease start with a three-month supportive periodontal treatment schedule. Failure to refer patients for recall sessions will cause recurrence of the disease.

COVID-19 lowers oral hygiene habits and dental visits due to its psychological effects on the patients, facemasks, social distancing,^8^ and decreased immune system competency, paving the way for the induction or exacerbation of periodontal diseases. This might result in a tsunami of periodontal diseases after the pandemic.
